# Elbow flexion recovery after intercostal nerve transfer in elderly patients: a clinical experience report

**DOI:** 10.3389/fsurg.2026.1776885

**Published:** 2026-04-08

**Authors:** Evelina Llorian, Gabriela Magalhães, Ingrid Espíndola, Fernando Guedes

**Affiliations:** Department of Surgery, Division of Neurosurgery, Gaffrée and Guinle University Hospital, School of Medicine, Federal University of Rio de Janeiro State (UNIRIO), Rio de Janeiro, Brazil

**Keywords:** brachial plexus injury, elbow flexion, elderly patients, intercostal nerve, nerve transfer

## Abstract

**Introduction:**

Restoration of elbow flexion is a primary goal in the surgical management of complete traumatic brachial plexus injuries (BPIs). When proximal donor nerves are unavailable, intercostal nerve (ICN) transfer to the musculocutaneous nerve (MCN) represents a well-established reconstructive option. However, elderly patients are markedly underrepresented in published series, and outcomes in this population remain poorly defined. The objective of this study was to evaluate clinical outcomes of ICN-to-MCN transfer in elderly patients and to identify perioperative factors associated with meaningful functional recovery.

**Methods:**

A retrospective case series was conducted. Over a 30-year period, four consecutive patients aged over 60 years who underwent ICN-to-MCN transfer for complete traumatic brachial plexus avulsion were identified. Demographic characteristics, surgical timing, coaptation strategy, and functional outcomes were analyzed.

**Results:**

Elderly patients accounted for less than 1.6% of more than 250 ICN-to-MCN transfers performed during the study period. All patients were male and sustained complete brachial plexus injuries following motorcycle accidents. One patient who underwent early reconstruction within 2 months of trauma, allowing direct neurorrhaphy without grafting, achieved useful elbow flexion (M4). This patient demonstrated preserved muscle bulk, normal testosterone levels, and strong adherence to postoperative motor rehabilitation. In contrast, delayed surgery beyond 5 months, particularly when nerve grafts were required, resulted in limited or absent recovery (M0–M2).

**Conclusions:**

ICN-to-MCN transfer remains a viable reconstructive option in carefully selected elderly patients. Favorable outcomes are influenced by modifiable factors, particularly earlier reconstruction, feasibility of tension-free coaptation, preserved muscle quality, and structures postoperative rehabilitation. While chronological age alone should not be considered a contraindication, the therapeutic window for successful nerve transfer is substantially narrower in older individuals.

## 1 Introduction

Restoring elbow flexion remains a fundamental goal in the surgical treatment of extensive brachial plexus injuries (BPIs), as it directly impacts independence in daily activities. When proximal donor options are absent, as often occurs in complete brachial plexus injuries, intercostal nerves (ICNs) become a valuable and accessible source for motor reinnervation. As nerve reconstruction techniques have evolved over the past decade, ICNs to musculocutaneous nerves (MCN) have consistently demonstrated its ability to reestablish useful biceps contraction (1–3).

The concept of nerve transfer in complete BPI avulsions dates back to Tuttle, who first described the use of cervical donor nerves for reinnervation (4). Subsequent pioneers such as Yeoman, Seddon, and Kotani documented the initial use of ICNs as donor nerves in the mid-20th century (5–7). In 1963, Seddon attempted ICN-to-MCN transfer using an intervening ulnar nerve graft, although results were limited due to the long regeneration distance and graft-induced axonal loss (6, 8, 9). Technical refinements followed, including direct coaptation techniques introduced by Tsuyama, Hara, and Kotani, later popularized by Millesi, Narakas, Terzis, Gu, and others (9–18). By expanding the distal dissection of ICNs toward the sternum, these authors achieved sufficient donor length to enable direct suture to the biceps motor branch, demonstrating superior functional recovery compared with graft-dependent approaches (14, 17–19).

Although the technique is widely accepted, managing these challenging cases often demands individualized choices. The surgeon must determine how many ICNs to harvest, whether a graft is necessary, and how long reconstruction can reasonably be delayed, decision shaped by anatomical constraints, available donors, and the goals set for each patient (20, 21).

Data from large clinical series indicate that age is a major predictor of functional outcomes after brachial plexus reconstruction, including ICN-to-MCN transfer. Patients younger than 30–40 years achieve significantly higher rates of functional elbow flexion compared with older individuals, who show a progressive decline in reinnervation potential and motor strength recovery (22–26). Despite this trend, elderly patients remain markedly underrepresented in the literature, and outcomes in those older than 50 years are rarely analyzed separately, leaving the specific influence of aging poorly defined in this context.

Given that many older adults remain active and motivated to undergo reconstructive procedures following traumatic injuries, there is a growing need for evidence that better reflects this population. Addressing this critical gap in the literature forms the central motivation of the present study: to understand how patients aged 60 years and older respond to ICN transfer for elbow flexion restoration, and to explore which perioperative factors may favor a meaningful recovery in this growing but understudied group.

## 2 Methods

This study is a retrospective, consecutive case series conducted at the Division of Neurosurgery of Gaffrée and Guinle University Hospital (HUGG), Federal University of the State of Rio de Janeiro, Brazil. The series includes patients treated both at HUGG and in the private practice setting, following the same surgical indications, operative techniques, and postoperative rehabilitation protocols.

Institutional approval was obtained from the HUGG Research Ethics Committee, which waived the need for study-specific informed consent due to the retrospective nature of the analysis. All patients had previously provided written informed consent for surgical treatment, including authorization for the use of anonymized clinical data and intraoperative images for scientific and education purposes.

The study followed the Preferred Reporting of Case Series in Surgery (PROCESS) guidelines.

### 2.1 Patient selection

Between 1995 and 2024, more than 250 IC-to-MCN transfers were performed at our institution. Among these, only four patients were aged ≥60 years and were therefore included in this study (less than 1.6% of all reconstructions). All patients were males, sustained complete traumatic brachial plexus avulsions injuries from motorcycle accidents, and presented with right-sided injuries (Tables 1, 2). None presented associated traumatic brain injury, or a prior history of neurodegenerative conditions or dementia. Patients with penetrating trauma, or central neurological impairment were excluded.

**Table 1:** Eligibility criteria for patient selection.

**Inclusion Criteria:**
Age ≥60 years Complete traumatic brachial plexus injury ICN-to-MCN nerve transfer No signs of spontaneous reinnervation prior surgery (ENMG confirmed) Minimum postoperative follow-up of 24 months
**Exclusion Criteria:**
Penetrating trauma injuries Associated traumatic brain injury History of neurodegenrative disease or dementia Severe comorbidities compromising postoperative rehabilitation or outcome assessment Prior reconstructive procedures for brachial plexus injury

**Table 2:** Clinical characteristics, surgical details, and functional outcomes of elderly patients undergoing ICN-to-MCN nerve transfer.

Patient	Age	Side	Injury mechanism	Brachial plexus Injury	Associated brain trauma	Delay to surgery (months)	Graft used	Additional nerve transfer	Elbow flexion (BMRC)	Outcomes
1	62	Right	Motorcycle accident	Complete BPI	No	2	No	XI → SSN	M4	Favorable
2	60	Right	Motorcycle accident	Complete BPI	No	5	Yes	XI → SSN	M0	Unfavorable
3	61	Right	Motorcycle accident	Complete BPI	No	5	No	XI → SSN	M0	Unfavorable
4	63	Right	Motorcycle accident	Complete BPI	No	5	No	XI → SSN	M2	Moderate

ICN, Intercostal nerve; MCN, Musculocutaneous nerve; XI → SSN, Spinal accessory nerve to suprascapular nerve transfer; BMRC, British Medical Research Council grading scale.

### 2.2 Preoperative evaluation

Clinical examination and electroneuromyography (ENMG) confirmed complete upper-limb paralysis with no signs of spontaneous reinnervation. Biceps muscle exhibited severe denervation changes in all cases. Pre-operative imaging (MRI) was used to confirm the presence of root avulsions. Time from trauma to surgery, presence of muscle atrophy, and associated reconstructive procedures were recorded for each patient.

### 2.3 Surgical technique

A submammary incision was performed along the fifth rib from the anterior axillary line toward the sternum, in all cases. The third and fourth ICNs were identified, carefully dissected along their course, and divided distally to maximize nerve length.

A second incision was made along the medial aspect of the upper arm to expose the MCN and its motor branch to the biceps branchii. The harvested ICNs were then tunneled subcutaneously toward the arm through a protected path. Nerve coaptation was carried out either to the musculocutaneous trunk or directly to the biceps motor branch, depending on the operative findings.

**Figure 1:**
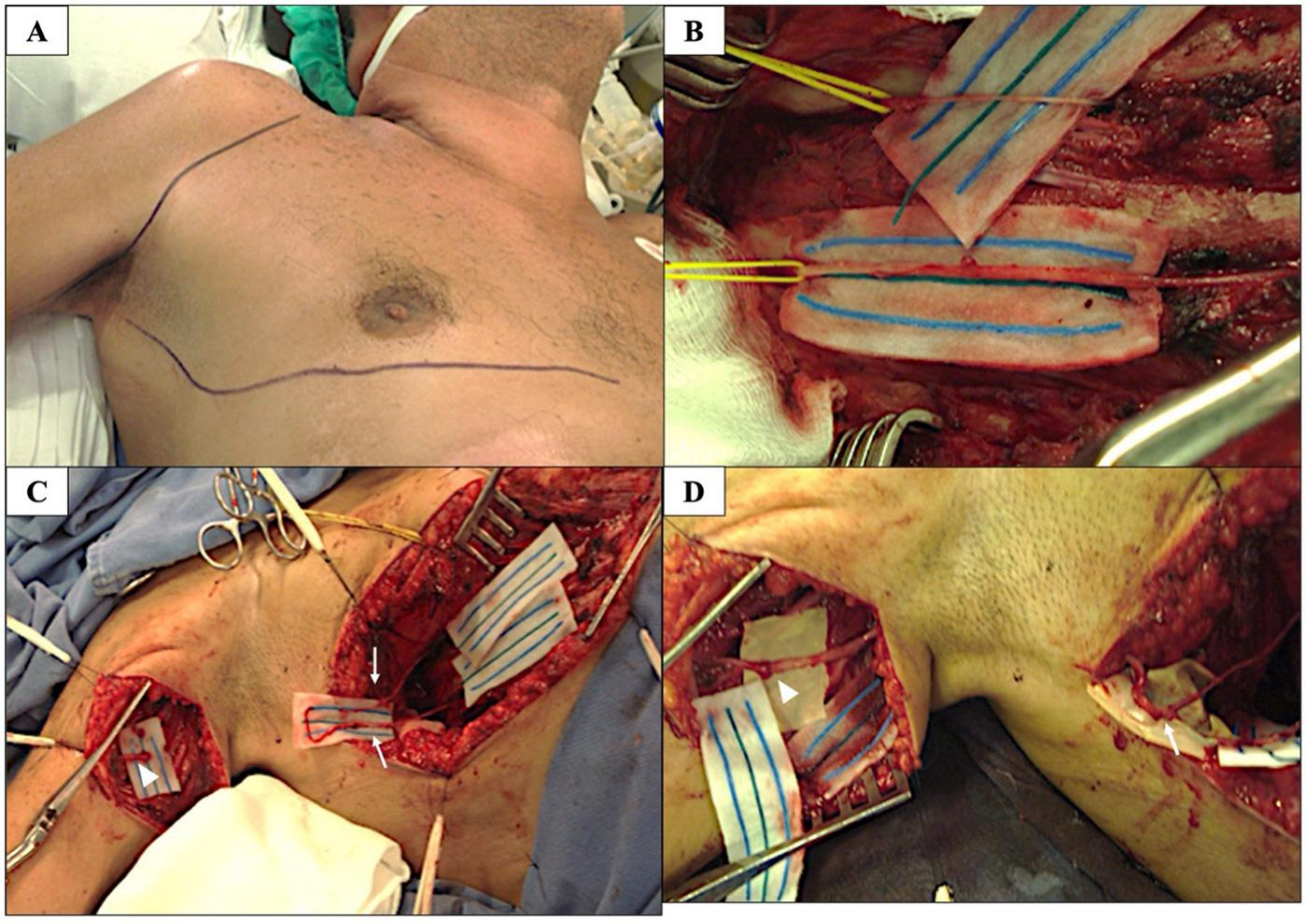
Surgical steps of the ICN-to-MCN transfer in a patient with favorable functional outcome (case 1). Images correspond to a 62-years-old male patient with favorable recovery of elbow flexion (MRC grade 4). **(A)** Right sided submammary incision performed along the fifth rib from the anterior axillary line toward the sternum. An additional infraclavicular incision was prepared to allow proximal extension if required. A second incision along the medial aspect of the upper arm was used to expose the musculocutaneous nerve (MCN) and its motor branch to the biceps brachii. **(B)** The third and fourth ICN after dissection, individually isolated and looped with yellow vessel loops. **(C)** Anatomical dissection of the third and fourth ICN (*white arrows*) and exposure of the MCN (*white arrowhead*) with identification of its motor branch to the biceps brachii. **(D)** Subcutaneous tunneling and end-to-end neurorrhaphy of the ICNs (*white arrow*) to the motor branch of the biceps muscle of the MCN (*white arrowhead*). All intraoperative images are anonymized and published with prior patient consent.

**Figure 2:**
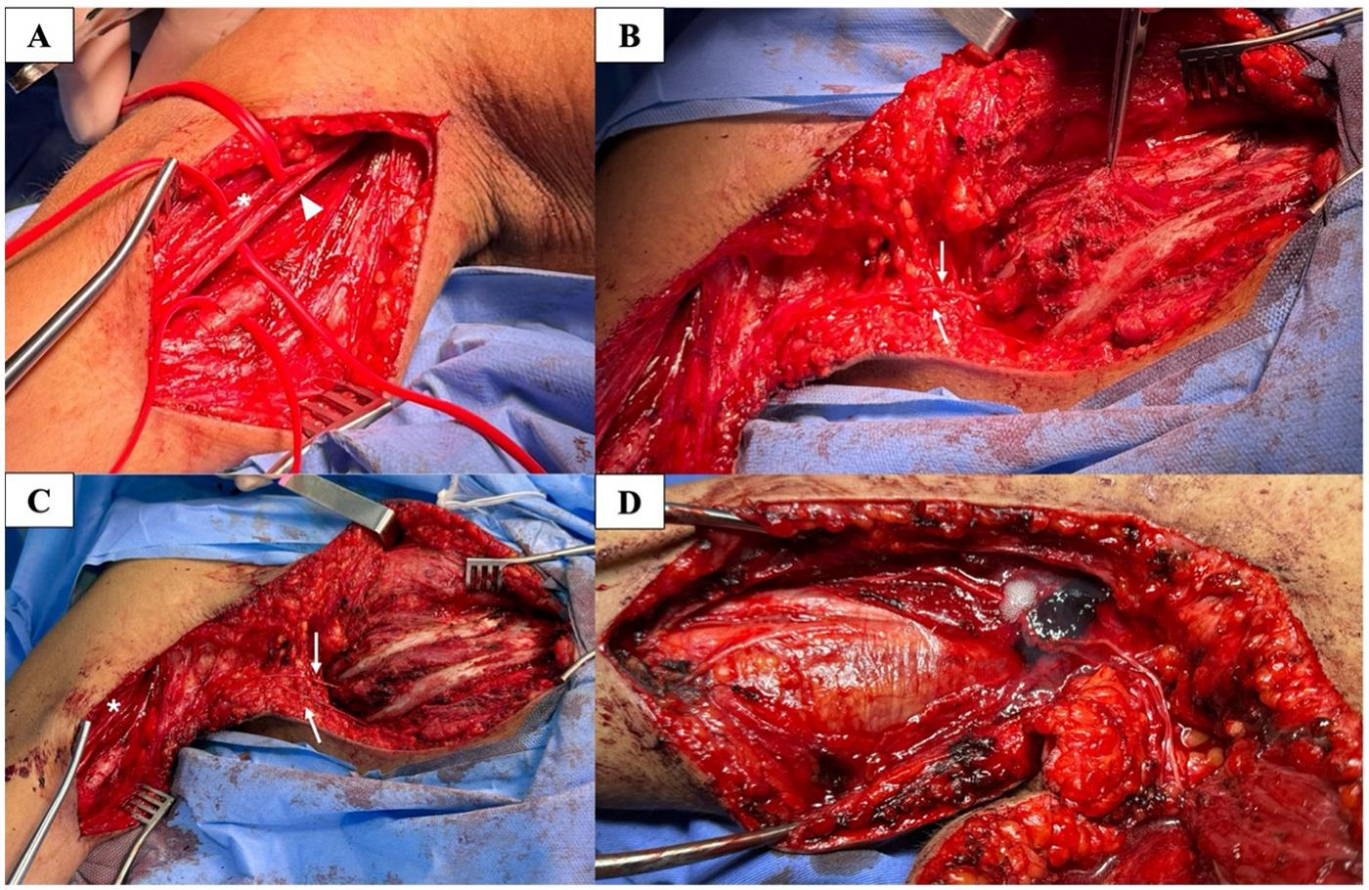
Intercostal-to-Musculocutaneous nerve transfer (case 4). Images correspond to Case 4, a 63-years-old male patient with partial functional recovery (MRC grade 2). **(A)** Medial aspect of the right upper arm showing the musculocutaneous nerve (MCN) and its motor branch to the biceps branchii. Intraoperative nerve stimulation showed no motor response. White arrowhead: MCN; asterisk; biceps motor branch. **(B)** Submammary incision along the fifth rib with expose of the intercostal nerves (ICNs) at the third and fourth rib levels. White arrows indicate the ICNs. **(C)** Subcutaneous tunneling of the ICNs toward the upper arm, enabling graft-free nerve transfer. White arrows: third and fourth ICNs; asterisk: biceps motor branch. **(D)** End-to-end coaptation of the ICNs to the biceps motor branch of the MCN usings 9-0 nylon sutures reinforced with fibrin glue. All intraoperative images are anonymized and published with prior patient consent.

A proximal epineurotomy of the MCN was performed to identify the biceps motor branch at its origin, enabling a shorter and tension-free neurorrhaphy and optimizing the biomechanical environment for axonal regeneration. In all cases, only the third and fourth ICNs were harvested and used as donor nerves, based on anatomical characteristics observed in this patient population. Whenever a direct coaptation was not feasible, interposition of nerve grafts were used to achieve a tension-free neurorraphy (Figures 1, 2).

In addition to the ICN-to-MCN transfer, all patients underwent a concomitant spinal accessory nerve (SAN) to suprascapular nerve (SSN) transfer as part of the reconstructive strategy aimed at improving shoulder stabilization and abduction. This procedure was performed with direct end-to-end coaptation whenever feasible.

All sutures were performed under operating microscopy using 9-0 nylon, reinforced with fibrin glue. Intraoperative nerve stimulation (Stimuplex® HNS12, B. Braun) was used throughout the procedure.

### 2.4 Postoperative management and follow-up

All patients began standardized rehabilitation twice weekly, focusing on passive and active elbow flexion and respiratory-motor coupling techniques. Follow-up visits occurred at 1, 6, 12, and 24 months postoperatively, with additional sessions as clinically indicated. Functional recovery was assessed using the British Medical Research Council (BMRC) scale. Outcomes were classified as favorable (BMRC 4–5), moderate (BMRC 2–3), or unfavorable (BMRC 0–1).

## 3 Results

Over a 30-year period, more than 250 ICN-to-MCN nerve transfers were performed at out institution; however, only four patients (less than 1.6%) were aged older than 60 years, highlighting the rarity of such reconstructions in elderly individuals. All four were male, sustained complete right-sided brachial plexus injury following motorcycle accidents, and presented without traumatic brain injury, or pre-existing neurodegenerative conditions.

Although each patient underwent transfer of the third and fourth ICNs, surgical timing and coaptation strategy differed among cases. The only patient treated within 2 months of the trauma, in whom direct coaptation was achieved without grafting, recovered useful elbow flexion (MRC grade M4). Notably, this patient had preserved muscle bulk and a long-standing history of resistance training prior to injury, and normal age-adjusted testosterone levels while under urological follow-up and testosterone replacement therapy. Among the patients who underwent reconstruction after approximately 5 months, one achieved limited recovery (M2) without the need for nerve grafting and likewise under urological follow-up and hormone replacement therapy with testosterone levels within the normal range of age. In contrast, the remaining two patients demonstrated no reinnervation (M0), one of whom required interposition nerve grafting.

At 24 months, functional outcomes were classified as favorable in one case, moderate in one, and unfavorable in two cases. These results result in substantial variability in recovery but consistently suggest that delay beyond the early post-injury period and the need for grafting markedly diminish the likelihood of achieving functional biceps reinnervation in older adults.

## 4 Discussion

Since the first intercostal nerve transfer described by Yeoman, Seddon, and Kotani, refinements in donor nerve dissection and direct coaptation techniques have progressively improved outcomes in the treatment of traumatic BPIs (3–7). These advances have reduced regeneration distance and axonal loss, establishing ICN-to-MCN transfer as a reliable option for the restoration of elbow flexion, particularly in younger adults (14–16). However, evidence regarding older patients remains limited, and functional expectations for individuals over 60 years of age remain poorly defined.

In most published series, ICN-to-MCN reconstruction involves two to four donor nerves, with the final number tailored to patient characteristics and surgical strategy (25, 26). In elderly patients, however, age-related anatomical and histological changes, such as reduced effective nerve caliber, lower fascicular density, and decreased tolerance to donor-site morbidity, may limit the feasibility or benefit of harvesting a higher number of ICNs. In this context, the deliberate use of two ICNs in our series represents a balanced approach aimed at achieving meaningful reinnervation while minimizing donor-related risk.

Our series provides rare clinical insight into this understudied age group. All patients sustained complete brachial plexus avulsion injuries and underwent transfer of two ICNs. Functional outcomes were heterogeneous and appeared strongly influenced by modificable surgical factors, particularly timing of reconstruction and the need for nerve grafting. The only patient who recovered useful meaningful elbow flexion (M4) underwent early reconstruction, two months after trauma, with direct coaptation and no interposition grafts, in the setting of preserved neuromuscular reserve. In contrast, reconstruction performed at longer intervals from trauma, particularly when interposition grafts were required, were associated with limited or absent reinnervation (M0–M2).

Given the apparent influence of nerve grafting on functional outcomes in our series, it is important to clarify that its use was not a technical limitation, but rather a deliberate, biologically driven decision. In elderly patients, ICNs frequently exhibit reduced effective diameter, a potential decrease in the number of functional axons, and lower tolerance to tension. Under these conditions, interposition grafts were favored to achieve a truly tension-free coaptation with optimal fascicular alignment. Although extensive mobilization of the MCN may technically allow direct coaptation, such maneuvers can introduce additional tension or compromise vascular supply, factors that are generally less well tolerated in older patients. Therefore, nerve grafting was selected to optimize biomechanical conditions for axonal regeneration and to respect age-related biological constraints, rather than as a consequence of limited exposure or surgical access.

Importantly, the favorable outcome occurred in a patient with preserved muscle bulk, a long-standing history of resistance training prior to injury, normal age-adjusted testosterone levels, and absence of associated traumatic brain injury. In addition, strict adherence to a structured postoperative motor rehabilitation program likely contributed to maintaining muscle trophism, optimizing neuromuscular activation, and facilitating functional integration of regenerating axons. While no causal inference can be drawn from a single case, these clinical characteristics suggest the presence of a more permissive biological substrate for reinnervation and functional recovery in selected elderly individuals.

Notably, although the patient who achieved partial recovery (M2) was under urological follow-up and receiving testosterone replacement therapy with age-adjusted range hormone levels within the normal range, the lack of substantial premorbid muscle bulk and long-term resistance training may have limited functional recovery. This suggest that hormonal normalization alone is unlikely to fully compensate for the combined effects of delayed reinnervation and reduced muscular reserve.

From a biological perspective, it is strongly associated with declining androgen levels, particularly testosterone in elderly male patients, and shows a clear inverse correlation with muscle mass, strength, and regenerative capacity (17–19). Lower levels of boths total and specifically free testosterone are consistently linked to greater muscle atrophy, impaired physical performance, and increased susceptibility to frailty. Testosterone exerts a direct anabolic effect on skeletal muscle through androgen receptors, promoting protein synthesis, and suppressing catabolic pathways (27–29). Consequently, age-related androgen deficiency creates a biologically less favorable substrate for muscle muscle preservation and recovery following nerve injury, particularly in the setting of chronic denervation.

These findings align with the known biological vulnerability of aging neuromuscular systems. Prolonged denervation accelerates irreversible changes, including motor neuron loss, Schwann-cell dysfunction, and degeneration of the neuromuscular junction, all of which substantially limit the potential for functional recovery. Although advanced age is a recognized prognostic factor, large clinical series demonstrate a gradual rather than absolute decline in strength recovery with increasing age (20–26, 30–32). Therefore, chronological age alone should not be considered a contraindication to nerve transfer.

Rather, our results highlight that the therapeutic window becomes significantly narrower in elderly patients. Favorable outcomes are more likely when surgery occurs early after trauma, muscle quality is preserved, direct coaptation is feasible, and systemic and cognitive health support postoperative neural plasticity. Conversely, prolonged denervation, severe muscle atrophy, and the need for extensive nerve grafting markedly reduce the likelihood of meaningful elbow flexion recovery.

When early referral is not possible, or when long regeneration distance necessitates nerve grafting, free functional muscle transfer may offer more predictable outcomes than nerve transfer alone in this population. Importantly, the extremely low number of elderly patients undergoing brachial plexus reconstruction at our center over three decades suggests that misconceptions regarding recovery potential and delayed referral continue to limit access to surgical treatment. Expanding awareness of realistic outcomes, aligning decision-making with patients goals, and promoting timely referral may help ensure that motivated older adults are not denied opportunities for functional restoration.

Although based on a small retrospective cohort, the present study represents a rare and valuable contribution, given the limited number of elderly individuals who undergo ICN-to-MCN reconstruction. These observations provide a foundation for refining patient selection, optimizing surgical timing, and guiding prognostic expectations in this growing population. Future multicenter collaboration and standardized reporting will be essential to better define long-term recovery trajectories in elderly patients with brachial plexus injuries.

## 5 Conclusions

Intercostal-to-musculocutaneous nerve transfer remains a viable reconstructive option for carefully selected elderly patients with complete traumatic brachial plexus avulsions injuries. Although functional recovery is generally less predictable than in younger populations, our findings suggest that outcomes are strongly influenced by modifiable surgical and biological factors. Reconstruction performed early in the post-traumatic period, particularly when direct, tension-free coaptation is feasible, and in the presence of preserved muscle quality, adequate neuromuscular reserve, and consistent adherence to structured postoperative rehabilitation appears to offer the greatest likelihood of achieving meaningful elbow flexion.

These results highlight that chronological age alone should not be considered an absolute contraindication to nerve transfer. Instead, timely referral, individualized surgical planning, and realistic prognostic counseling are essential to optimize outcomes. Strengthening referral pathways and improving awareness of achievable goals may help ensure that motivated older adults are not excluded from reconstructive opportunities solely on the basis of age.

## Data Availability

The original contributions presented in the study are included in the article/Supplementary Material, further inquiries can be directed to the corresponding author.

## References

[1] Chuang DC. Nerve transfers in adult brachial plexus injuries: my methods. Hand Clin. (2005) 21(1):71–82. 10.1016/j.hcl.2004.10.004 15668067

[2] Malessy MJ Thomeer RT. Evaluation of intercostal to musculocutaneous nerve transfer in reconstructive brachial plexus surgery. J Neurosurg. (1998) 88(2):266–71. 10.3171/jns.1998.88.2.0266 9452234

[3] Seddon HJ. Nerve grafting. Ann R Coll Surg Engl. (1963) 32(5):269–80. 13987582 PMC2311558

[4] Tuttle HK. Exposure of the brachial plexus with nerve transplantation. JAMA. (1913) 61:15–7. 10.1001/jama.1913.04350010017006

[5] Yeoman P Seddon H. Brachial plexus injuries: treatment of the flail arm. J Bone Joint Surg. (1961) 43B:493–500. 10.1302/0301-620X.43B3.493

[6] Seddon HJ. Nerve grafting. J Bone Joint Surg. (1963) 45B:447–61. 10.1302/0301-620X.45B3.447 14058318

[7] Kotani P Matsuda H Suzuki T. Trial surgical procedures of nerve transfers to avulsion injuries of plexus brachialis. Exerpta Medica Inter Congress Series. (1972) 291:348.

[8] Goubier JN Teboul F. Transfer of the intercostal nerves to the nerve of the long head of the triceps to recover elbow extension in brachial plexus palsy. Tech Hand Up Extrem Surg. (2007) 11(2):139–41. 10.1097/bth.0b013e31803105e1 17549019

[9] Sedel L. The results of surgical repair of brachial plexus injuries. J Bone Joint Surg Br. (1982) 64:54Y66. 10.1302/0301-620X.64B1.7068721 7068721

[10] Dutton RO Dawson EC. Elbow flexorplasty: an analysis of long-term results. J Bone Joint Surg. (1981) 63:1064–9. 10.2106/00004623-198163070-00003 7276043

[11] Mayer L Green W. Experiences with the steindler flexorplasty at the elbow. J Bone Joint Surg. (1954) 36:775–89. 10.2106/00004623-195436040-00009 13174607

[12] Carroll RE Hill NA. Triceps transfer to restore elbow flexion: a study of fifteen patients wtih paralytic lesions and arthrogryposis. J Bone Joint Surg. (1970) 52:239–44. 10.2106/00004623-197052020-00004 5440001

[13] Friedman AH Nunley JA Goldner RD Oakes WJ Goldner JL Urbaniak JR. Nerve transplantation for the restoration of elbow flexion following brachial plexus avulsion injuries. J Neurosurg. (1990) 72:59–64. 10.3171/jns.1990.72.1.0059 2294186

[14] Kanaya F Gonzalez M Punk CM Kutz J Kleinert HE Tsai TM. Improvement in motor function after brachial plexus surgery. J Hand Surg. (1990) 15A:30–6. 10.1016/S0363-5023(09)91101-7 2299164

[15] Wood MB. Nerve Repair and Reconstruction. in: Atlas of Reconstructive Microsurgery. Rockville, MD: Aspen Publications (1990). p. 32–4.

[16] Millesi H. Surgical management of brachial plexus injuries. J Hand Surg. (1977) 2:367–79. 10.1016/S0363-5023(77)80046-4 198459

[17] Borst SE. Interventions for sarcopenia and muscle weakness in older people. Age Ageing. (2004) 33(6):548–55. 10.1093/ageing/afh201 15385272

[18] Roy TA Blackman MR Harman SM Tobin JD Schrager M Metter EJ. Interrelationships of serum testosterone and free testosterone index with FFM and strength in aging men. Am J Physiol Endocrinol Metab. (2002) 283(2):E284–94. 10.1152/ajpendo.00334.2001 12110533

[19] Guedes F Llorian E Henriques VM Haikal N Sanches GE. Outcomes of oberlin transfer in elderly patients: a case series. World Neurosurg. (2025) 194:123510. 10.1016/j.wneu.2024.11.093 39603458

[20] Tsuyama N Hara T. Reconstructive surgery for traumatic brachial plexus injuries. Clin Orthop Surg. (1968) 3:675–87.

[21] Lykissas MG Kostas-Agnantis IP Korompilias AV Vekris MD Beris AE. Use of intercostal nerves for different target neurotization in brachial plexus reconstruction. World J Orthop. (2013) 4(3):107–11. 10.5312/wjo.v4.i3.107 23878776 PMC3717241

[22] Celli L Rovesta C Balli A. Neurotization of brachial plexus avulsion with intercostal nerves (personal techniques). In: Brunelli G, editor. Textbook of Microsurgery. Milano: Masson (1988). p. 789–95.

[23] Merrell GA Barrie KA Katz DL Wolfe SW. Results of nerve transfer techniques for restoration of shoulder and elbow function in the context of a meta-analysis of the English literature. J Hand Surg Am. (2001) 26(2):303–14. 10.1053/jhsu.2001.21518 11279578

[24] Leland HA Azadgoli B Gould DJ Seruya M. Investigation into the optimal number of intercostal nerve transfers for musculocutaneous nerve reinnervation: a systematic review. Hand (N Y). (2018) 13(6):621–6. 10.1177/1558944717744280 29185810 PMC6300187

[25] Cho AB Iamaguchi RB Bersani Silva G Paulos RG Kiyohara LY Sorrenti L Intercostal nerve transfer to the biceps motor branch in complete traumatic brachial plexus injuries. Microsurgery. (2015) 35(6):428–31. 10.1002/micr.22453 26202174

[26] de Mendonça Cardoso M Gepp R Lima FL Gushiken A. Intercostal to musculocutaneous nerve transfer in patients with complete traumatic brachial plexus injuries: case series. Acta Neurochir (Wien). (2020) 162(8):1907–12. 10.1007/s00701-020-04433-3 32506331

[27] Shigehara K Kato Y Izumi K Mizokami A. Relationship between testosterone and sarcopenia in older-adult men: a narrative review. J Clin Med. (2022) 11(20):6202. 10.3390/jcm11206202 36294523 PMC9605266

[28] Yuki A Otsuka R Kozakai R Kitamura I Okura T Ando F Relationship between low free testosterone levels and loss of muscle mass. Sci Rep. (2013) 3:1818. 10.1038/srep01818 23660939 PMC6504823

[29] LeBlanc ES Wang PY Lee CG Barrett-Connor E Cauley JA Hoffman AR Higher testosterone levels are associated with less loss of lean body mass in older men. J Clin Endocrinol Metab. (2011) 96(12):3855–63. 10.1210/jc.2011-0312 21976718 PMC3232620

[30] Bhatia A Kulkarni A Zancolli P Martinez RR Clifton J El-Gammal T The effect of age and the delay before surgery on the outcomes of intercostal nerve transfers to the musculocutaneous nerve: a retrospective study of 232 cases of posttraumatic total and near-total brachial Plexus injuries. Indian J Plast Surg. (2020) 53(2):260–5. 10.1055/s-0040-1716081 32884192 PMC7458838

[31] Socolovsky M di Masi G Bonilla G Lovaglio AC López D. Age as a predictor of long-term results in patients with brachial Plexus palsies undergoing surgical repair. Oper Neurosurg. (2018) 15(1)):15–24. 10.1093/ons/opx184 28961945

[32] Sharma R Gaba S Modi M. Age correlation in upper brachial plexus injury patients undergoing nerve transfer surgeries. Brain Spine. (2022) 2:101695. 10.1016/j.bas.2022.101695 36506296 PMC9729817

